# Autonomic Dysfunction, Psychosocial Factors, and Pain Sensitivity in Persistent Spinal Pain: A Systematic Review

**DOI:** 10.3390/jcm15145571

**Published:** 2026-07-16

**Authors:** Mahiques-Sanchis Alex, Alonso-Martín Mónica, Martínez-Soler Marta, Belda-Antolí Mariola, Benlloch-García Agustín, Huertas-Ramirez Borja, Yeste-Fabregat Mireia, Vicente-Mampel Juan

**Affiliations:** 1 Doctoral School, Catholic University of Valencia San Vicente Martir, 46001 Valencia, Spain; alex.mahiques@ucv.es (M.-S.A.); marta.martinez@ucv.es (M.-S.M.); agustin.benlloch@ucv.es (B.-G.A.); borja.huertas@ucv.es (H.-R.B.); 2 Department of Physiotherapy, Medicine and Health Science School, Catholic University of Valencia, 46001 Valencia, Spain; mariola.belda@ucv.es (B.-A.M.); mireia.yeste@ucv.es (Y.-F.M.); juan.vicente@ucv.es (V.-M.J.)

**Keywords:** persistent spinal pain, heart rate variability, pressure pain threshold, psychosocial factors, autonomic nervous system, quantitative sensory testing

## Abstract

**Background/Objectives**: Persistent spinal pain, including chronic low back pain (CLBP), chronic neck pain, and whiplash-associated disorders (WAD), is a highly prevalent musculoskeletal condition. The recent literature emphasizes a biopsychosocial framework, acknowledging that altered pain processing, psychological impairments, and autonomic nervous system dysfunction are associated with chronic pain. However, the concurrent within-sample relationships between these domains remain poorly understood and inconsistently reported. To systematically evaluate the associations among physiological autonomic measures, validated psychosocial scales, and pressure algometry in adults with persistent spinal pain. **Methods:** A systematic search was conducted in accordance with PRISMA guidelines. A systematic search was conducted across four electronic databases (PubMed, Web of Science, Scopus, and CINAHL) for studies evaluating all three domains within the same cohort. The search was restricted to peer-reviewed articles published in English and Spanish. Two independent blinded reviewers screened and selected the studies. Eligible studies evaluated within-sample relationships across three domains: autonomic indices (e.g., heart rate variability (HRV) and blood pressure (BP)), psychosocial factors (e.g., kinesiophobia and pain catastrophizing), and quantitative sensory testing (QST) (e.g., pressure pain threshold (PPTs)). Quality assessment was performed using the National Institutes of Health (NIH) tools. Due to significant methodological heterogeneity, the data were synthesized narratively. **Results:** Six studies fulfilled the eligibility criteria. The narrative synthesis revealed a preliminary pattern showing that objective functional outcomes and experimental pain processing tend to show closer associations with autonomic proxies (such as heart rate variability), whereas subjective clinical outcomes (such as perceived disability) are more consistently linked to psychosocial factors (such as kinesiophobia and catastrophizing). **Conclusions:** These preliminary findings suggest potentially distinct domain-specific associations rather than a uniform pathophysiological mechanism. Future specialized research and research-informed practice should incorporate multimodal assessments to better characterize individual patient profiles.

## 1. Introduction

Persistent spinal pain conditions, including chronic low back pain (CLBP), chronic neck pain, and whiplash-associated disorders (WAD), are among the leading causes of disability worldwide and impose a substantial healthcare and socioeconomic burden [1,2,3]. These conditions are characterized by alterations in three interrelated domains—autonomic nervous system function, pain processing, and psychosocial factors—whose interactions may contribute to the considerable heterogeneity in clinical presentation and treatment response. Within this spectrum, Failed Back Surgery Syndrome (FBSS), increasingly referred to as Persistent Spinal Pain Syndrome type 2 (PSPS-T2), represents one of the most complex manifestations of chronic spinal pain. The term PSPS reflects the recognition that persistent pain after lumbar surgery cannot always be explained by structural abnormalities and better encompasses patients with and without previous spinal surgery [4,5,6,7]. Clinical outcomes are influenced by multiple biological, psychological, and social factors, including anxiety, depression, emotional state, self-esteem, sex, pain catastrophizing, kinesiophobia, and self-efficacy, all of which have been associated with disability and treatment response, particularly following spinal cord stimulation [8,9,10,11,12,13].

These findings support the biopsychosocial (BPS) model, which conceptualizes pain and disability as the result of dynamic interactions among biological, psychological, and social factors [14]. Patients with CLBP frequently present with high levels of functional disability, fear of movement, pain catastrophizing, and low self-efficacy, which significantly influence clinical progression and treatment response [15]. Since structural abnormalities alone rarely explain persistent spinal pain [16], current recommendations advocate combining patient-reported outcomes with objective clinical measures to guide assessment and treatment [17]. However, subjective questionnaires frequently show limited agreement with objective physiological findings, emphasizing the multidimensional nature of chronic pain. More recently, the biopsychosocial-enactive model has expanded this framework by considering pain as an embodied and context-dependent experience emerging through continuous interactions between the individual and their physical, social, and cultural environment [18]. This perspective recognizes patients as active agents in the construction of their pain experience and suggests that subjective disability and objective physiological regulation may represent distinct but complementary aspects of the adaptive response to persistent pain. Consequently, integrating physiological, psychological, and pain-processing assessments may provide a more comprehensive understanding of persistent spinal pain than evaluating each domain separately [19].

Among the biological mechanisms, autonomic nervous system dysfunction has received increasing attention. Heart rate variability (HRV) is widely used as a non-invasive indicator of autonomic regulation and has been associated with pain severity, impaired autonomic flexibility, psychological distress, and poorer clinical outcomes in chronic pain populations [20,21]. Resting blood pressure and heart rate have also been proposed as indirect markers of autonomic dysfunction, although they are influenced by several biological and behavioral factors [22]. In parallel, quantitative sensory testing (QST), including pressure pain thresholds (PPTs), conditioned pain modulation (CPM), and temporal summation, provides objective information on pain sensitivity and endogenous pain modulation, helping to identify mechanisms such as central sensitization [23,24]. Nevertheless, previous reviews have reported inconsistent associations between experimental pain measures and psychosocial variables, suggesting that these domains may represent different aspects of chronic pain [25,26,27]. Psychosocial factors, including pain catastrophizing, kinesiophobia, anxiety, depression, and central sensitization-related symptoms, are consistently associated with pain intensity, disability, and reduced quality of life [14,15,28,29]. However, because emotional responses involve behavioral, cognitive, and physiological dimensions that are not always concordant, self-reported questionnaires may not fully reflect the biological mechanisms underlying persistent pain [30,31]. Although autonomic regulation, psychosocial factors, and pain-processing abnormalities have each been extensively investigated, few studies have simultaneously examined their relationships within the same cohort of patients with persistent spinal pain. Consequently, it remains unclear whether these domains reflect a common pathophysiological mechanism or relatively independent but interacting dimensions of chronic pain [20,32].

Therefore, this systematic review aimed to synthesize the available evidence regarding the associations among autonomic physiological measures, validated psychosocial variables, and experimental pain-processing outcomes in adults with persistent spinal pain. Specifically, it sought to determine whether consistent cross-domain relationships exist among autonomic regulation, psychosocial factors, and quantitative sensory testing measures, thereby contributing to a more integrated understanding of persistent spinal pain and informing future research and personalized clinical assessment.

## 2. Materials and Methods

This systematic review was conceptualized and conducted in strict adherence to the Preferred Reporting Items for Systematic Reviews and Meta-Analyses (Supplementary Material PRISMA 2020 Checklist) and in accordance with the recommendations of the Cochrane Handbook for Systematic Reviews of Interventions [33,34].

### 2.1. Eligibility Criteria

Studies were eligible if they evaluated the concurrent relationship between autonomic, psychosocial, and pain processing variables within the same sample of individuals with spinal pain conditions. Furthermore, studies were restricted to those published in peer-reviewed journals in English or Spanish. The population of interest included adults (≥18 years) with spinal pain of at least 3 months’ duration, encompassing CLBP, persistent or recurrent neck pain, WAD, and clearly defined cohorts with persistent spinal pain syndrome, specifically clarifying that both PSPS type 1 (without prior surgery) and PSPS type 2 (with a history of spinal surgery) were eligible. Eligible studies were required to employ non-interventional assessments across three domains of interest within the same cohort: (1) physiological or autonomic variables, including measures such as HRV, resting HR or BP as autonomic proxies, and electrodermal or sympathetic indices (e.g., skin conductance or sympathetic skin response); (2) psychosocial variables assessed using validated self-report instruments relevant to pain, such as pain catastrophizing, kinesiophobia/fear of movement, central sensitization-related symptoms, or closely related psychological distress constructs; and (3) PPTs assessed using pressure algometry or QST, particularly PPTs or equivalent pressure pain measures. This standardized term (PPTs) was used consistently hereafter.

This review specifically focused on within-sample relationships among these three domains in individuals with persistent spinal pain, rather than between-group comparisons. This strict eligibility criterion, requiring all three domains within the same cohort, was methodologically mandatory to capture true, unconfounded within-sample cross-domain associations, thereby avoiding the profound population-level selection biases that would arise from synthesizing separate data across disparate cohorts. Furthermore, because autonomic activity, psychological states, and pain sensitivity exhibit substantial temporal fluctuations, studies were only eligible if all three domains were assessed concurrently during the same evaluation visit or within the same cross-sectional baseline assessment period. Accordingly, the primary outcomes of interest were quantitative associations (e.g., correlation or regression coefficients) between physiological or autonomic variables and psychosocial factors, as well as between physiological measures and PPTs, including integrative analyses examining relationships across the three domains in the same sample. It was explicitly recognized that parameters such as resting HR and BP are heavily influenced by multiple non-autonomic confounding factors, including medication use (e.g., beta-blockers), physical fitness, concurrent pain intensity, caffeine intake, acute anxiety, and underlying cardiovascular disease. Consequently, these measures were interpreted cautiously as clinical physiological proxies of the overall cardiovascular autonomic state rather than pure, isolated measures of sympathetic or parasympathetic tone.

Studies were excluded if they involved acute pain populations (<3 months), non-spinal pain populations in which spinal-specific data could not be separated, or if they did not simultaneously assess all three required domains within the same cohort. Studies focusing solely on between-group comparisons without examining within-sample associations were excluded. Additionally, purely interventional analyses without extractable baseline data, case reports, narrative reviews, editorials, grey literature, trial registries, and conference abstracts lacking peer-reviewed, sufficient quantitative data were excluded.

### 2.2. Information Sources and Search Strategy

A comprehensive and systematic literature search was performed in four major electronic databases: PubMed, Scopus, Web of Science, and CINAHL. The search strategy utilized a combination of Medical Subject Headings (MeSH) and free-text keywords, structured into four interconnected thematic blocks: (1) condition-specific terms (e.g., “chronic low back pain”, “whiplash-associated disorder”, “persistent spinal pain”); (2) physiological/autonomic metrics (e.g., “heart rate variability”, “blood pressure”, “skin conductance”); (3) psychosocial factors (e.g., “pain catastrophizing,” “kinesiophobia”, “psychological distress”); and (4) pain sensitivity measures (e.g., “pressure pain threshold”, “quantitative sensory testing”, “algometry”). Boolean operators (AND/OR) were used to link thematic blocks. The detailed search syntax tailored for each database is provided in Supplementary Table S1, and formal literature searches were executed and completed between January 2026 and February 2026.

### 2.3. Study Selection

Following the retrieval of articles from the targeted databases, all records were exported to a citation manager, where duplicates were removed. The selection process involved dual-stage screening. Initially, two independent investigators evaluated the titles and abstracts of the retrieved records according to the predefined eligibility criteria completely independently and in a blinded manner to each other’s decisions using specialized screening software Rayyan, web-based application, accessed March 2026 (Rayyan). Before the formal process, a calibration pilot testing session on a random sample of 10% of the retrieved studies was conducted to align the eligibility criteria. Articles deemed potentially relevant advanced to the second stage, in which full-text manuscripts were obtained and scrutinized, maintaining the same independent and blinded approach. In instances of persistent disagreement during either phase, formal arbitration was performed through consultation with a third and fourth independent reviewers (A. B. G. and M. Y. F.) to reach a definitive consensus.

### 2.4. Data Extraction

Data extraction was systematically performed using a standardized, custom-built extraction form. Two independent authors extracted the data using a predefined Excel spreadsheet (A. M. S. and M. A. M.). The extracted data were mapped to the following domains: (1) study characteristics (first author, publication year, study design, geographical location, population type); (2) demographic and clinical profiles of the population (sample size, age, sex distribution, body mass index, pain duration, and pain severity); (3) psychosocial assessments (for example, TSK, PCS, anxiety, depression, and disability indices such as NDI); (4) physiological/autonomic indices (for example, HRV frequency domains [LF, HF, LF/HF ratio], time domains [RMSSD, SDNN], resting HR, and BP); and (5) pain processing metrics (for example, PPTs values across localized and remote body sites, CPM, wind-up ratio, and QST measures such as MPT and MDT).

### 2.5. Risk of Bias Assessment

Quality assessment was performed using the National Institutes of Health (NIH) Quality Assessment Tool with design-specific checklists [35]. For observational cohort and cross-sectional studies, the domains included research question clarity, population definition, participation rate, selection criteria consistency, sample size justification, exposure and outcome measurement, assessor blinding, follow-up (for cohort studies), and confounding control. For case–control studies, the tool assessed case and control selection, population comparability, case definition, exposure assessment, blinding, and confounder adjustment. Certain domains were considered not applicable depending on the study design (e.g., follow-up-related items in cross-sectional studies and temporality in experimental paradigms in which exposure and outcome were measured concurrently). Each study was rated as good, fair, or poor overall, based on the tool guidance and reviewer judgment. Any disagreements in quality ratings were resolved through discussion and arbitration by a third reviewer when required.

### 2.6. Data Synthesis

Owing to the profound methodological, clinical, and statistical heterogeneity inherent in the included studies, we determined that conducting a quantitative meta-analysis would yield mathematically invalid and clinically misleading results. Consequently, data pooling was not possible in this study. Heterogeneity manifests in multiple dimensions. Clinically, the operationalization of autonomic proxies was highly divergent, with studies utilizing different domains of HRV, resting cardiovascular vitals, and skin conductance. Psychosocial constructs also varied widely, encompassing the TSK, PCS, HADS, and CHQ-12. Furthermore, PPTs were conducted across different anatomical landmarks depending on the specific spinal condition investigated. Statistically, the outcome reporting was heterogeneous, comprising an incompatible mix of Pearson/Spearman correlation coefficients, partial correlations, multiple regression beta values, and adjusted mean differences.

A narrative synthesis approach was used to address this variability. The findings were organized in tables and grouped based on the types of physiological measurements. To achieve a structured analysis, the direction (positive, negative, or non-significant) and specific statistical magnitude (correlation coefficients, regression weights, or effect sizes) of the cross-domain associations were extracted from each primary study. The consistency of these relationships was subsequently evaluated qualitatively by systematically comparing whether matching statistical directions and patterns recurred across different persistent spinal pain cohorts. This approach enabled a structured qualitative analysis of the direction, consistency, and strength of the relationships between autonomic function, psychosocial factors, and pain sensitivity in individuals with persistent spinal pain [36,37].

## 3. Results

### 3.1. Literature Search

**Figure 1:**
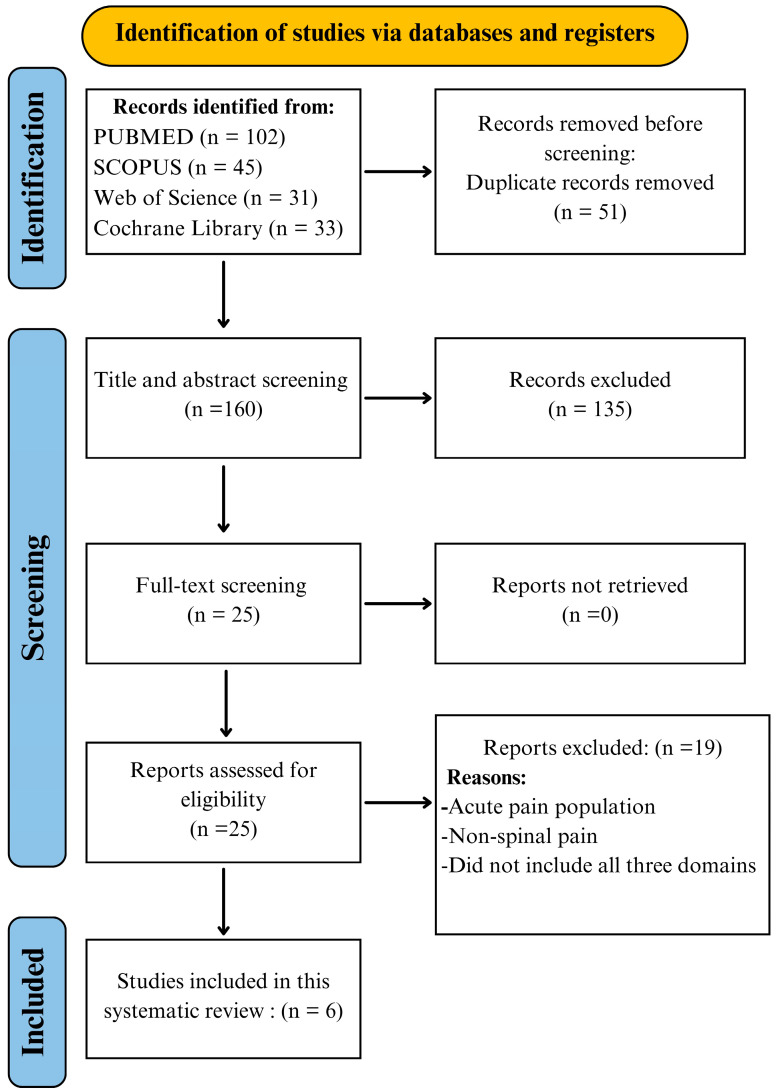
PRISMA flow diagram of the study selection process.

A total of 211 records were identified through the database search. After removing duplicates (n = 51), 160 records were screened, of which 25 full-text articles were assessed for eligibility, and six studies were finally included in this systematic review (Figure 1).

### 3.2. Characteristics of Included Studies

Six studies met the inclusion criteria and were included in this systematic review [20,32,36,37,38,39]. The baseline characteristics of the patients are shown in Table 1. The study designs comprised three cross-sectional studies [20,32,36], two case–control studies [38,39], and one observational prospective cohort study [37]. The studies were conducted in Brazil, Belgium, the Netherlands, Taiwan, and Australia.

The pooled sample size across all studies included 325 participants with persistent spinal pain. The specific clinical populations included those with CLBP, chronic neck pain, and WAD. The populations were predominantly female (ranging from 55.3% to 80.0%), with mean ages ranging broadly from 22.4 years in a younger neck pain cohort to 46.0 years in a CLBP cohort. Pain duration varied significantly across studies, ranging from a median of 26.4 months in an unoperated CLBP cohort to a mean of 65 months in a postsurgical group.

### 3.3. Quality Assessment

Based on the NIH Quality Assessment Tools (Supplementary Tables S2 and S3), the overall methodological quality was generally fair, with notable limitations. Only one study achieved an overall rating of “good”. The remaining five studies were rated as “Fair”. Common methodological limitations across these “Fair” studies included a lack of sample size justification, failure to measure exposure before outcomes (inherent to cross-sectional designs), and unblinded outcome assessors.

### 3.4. Bivariate Associations

#### 3.4.1. Autonomic and Psychosocial Associations

In a cross-sectional study of patients with chronic neck pain, Santos-de-Araújo et al. [36] identified a significant positive association between nonlinear HRV in the supine position (SD2/SD1 ratio) and pain catastrophizing, measured using the PCS (r = 0.384, *p* < 0.05; note that this reflects uncorrected *p*-values). Conversely, associations between psychosocial variables and other time- and frequency-domain HRV indices were generally weak and non-significant across all postural conditions in the present study. Furthermore, no significant association was observed between HRV measurements and kinesiophobia (*p* ≥ 0.05). In line with these findings, Bandeira et al. [39] reported no significant association between stress-induced HRV reactivity and psychosocial measures, including kinesiophobia, catastrophizing, anxiety, and depression. Crucially, it must be highlighted that De Kooning et al. [38] and White et al. [32] explicitly applied strict statistical corrections for multiple comparisons (Bonferroni and Holm methods, respectively); following these adjustments, both cohorts failed to identify meaningful relationships between resting autonomic measures and disability, posttraumatic stress symptoms, or pain-related psychological outcomes (Supplementary Table S4). Overall, evidence supporting consistent associations between autonomic regulation and psychosocial variables in chronic spinal pain populations is limited to date.

#### 3.4.2. Autonomic and Pain Processing Associations

In patients with chronic WAD, De Kooning et al. [38] identified significant associations between autonomic cardiovascular regulation and laboratory-based experimental pain processing. Based on uncorrected *p*-values, a higher resting HR was associated with higher local mechanical PPTs at the upper trapezius (r = 0.480, *p* = 0.008). In addition, markers of autonomic regulation were linked to descending pain inhibitory capacity, as greater CPM efficacy was associated with lower resting LF power and greater HRV reactivity during cuff inflation. Conversely, White et al. [32] failed to identify consistent associations between resting autonomic measures and any experimental pain-processing outcomes, including PPTs, temporal summation (WUR), and CPM efficacy, after applying rigorous corrections for multiple comparisons. Similarly, De Kooning et al. [38] reported no significant association between resting HR and experimental PPTs measured at remote anatomical sites (Supplementary Table S5). Overall, evidence regarding the relationship between autonomic regulation and experimental pain-processing measures in chronic spinal pain populations is inconsistent, with some findings suggesting localized links to descending pain modulation.

#### 3.4.3. Psychosocial and Pain Processing/Functional Associations

In patients with CLBP, Bandeira et al. [39] found that higher baseline kinesiophobia was significantly associated with greater clinical pain exacerbation following passive visualization of daily activities (r = 0.38; *p* = 0.009). Concurrently, longitudinal findings from Ansuategui Echeita et al. [37] demonstrated that reductions in central sensitization symptoms over a rehabilitation period were significantly associated with reductions in self-reported clinical disability (r = 0.44, *p* < 0.01). However, the associations between psychosocial variables and objective functional outcomes were weak and inconsistent. Bandeira et al. [39] reported no significant relationships between clinical pain exacerbation and catastrophizing, anxiety, or depression. Similarly, Ansuategui Echeita et al. [37] found that baseline and longitudinal changes in central sensitization symptoms were not significantly associated with objective functional performance measures, including lifting capacity, physical functioning, and work ability (Supplementary Table S6). Overall, psychosocial variables appeared to demonstrate stronger relationships with subjective clinical pain and disability outcomes than with objective functional performance outcomes.

#### 3.4.4. Associations Across Autonomic, Pain, and Disability Outcomes

Across the reviewed literature, autonomic measures have demonstrated more consistent statistical links when crossed with subjective clinical pain severity and disability indices than experimental laboratory testing. In a large cohort of patients with chronic neck pain, Kang et al. [20] reported significant negative associations between multiple HRV indices and self-reported clinical disability (NDI), indicating that lower autonomic variability was related to greater self-reported restriction. Concurrently, lower HRV is associated with higher clinical pain intensity and greater psychological distress. Comparable findings were reported by Santos-de-Araújo et al. [36], who observed significant associations between autonomic measures and movement-related clinical pain, disability, and pain chronicity in patients with chronic neck pain. In particular, vagally mediated and nonlinear HRV indices demonstrated inverse relationships with disabilities and movement-related pain. Additionally, frequency-domain HRV measures obtained while standing were associated with the resting clinical pain intensity.

From a longitudinal perspective, Ansuategui Echeita et al. [37] demonstrated that improvements in vagal tone (ΔRMSSD) were significantly associated with improvements in objective lifting capacity in patients with CLBP (r = 0.41, *p* < 0.01). However, not all cross-domain associations were consistent with this hypothesis. Kang et al. [20] found no significant association between the LF/HF ratio and disability, while White et al. [32] failed to identify meaningful relationships between resting cardiovascular measures and clinical pain intensity, disability, or pain duration in chronic WAD populations. Furthermore, Ansuategui Echeita et al. [37] reported no significant cross-sectional associations between baseline RMSSD and functional outcomes, and De Kooning et al. [38] found no relationship between resting HR and remote experimental PPTs measurements (Supplementary Table S7). Collectively, the evidence suggests that autonomic alterations, particularly reduced vagal modulation, are frequently linked to greater clinical pain severity and disability, although the findings remain heterogeneous across chronic spinal pain populations.

#### 3.4.5. Multivariable Integrative Associations

Two studies moved beyond bivariate analyses by applying multivariable regression models that integrated autonomic, psychosocial, and experimental pain processing variables to predict clinical and functional outcomes (Supplementary Table S8). In patients with CLBP, Bandeira et al. [39] found that psychosocial factors were the strongest predictors of clinical pain exacerbation during a visual stress task. Specifically, higher baseline kinesiophobia significantly predicted increased pain intensity, whereas autonomic baseline tone (LF/HF ratio) did not independently contribute to the model.

In parallel, Ansuategui Echeita et al. [37] demonstrated that different domains are associated with distinct aspects of functioning in populations with CLBP. Cross-sectionally, objective lifting capacity was primarily associated with experimental pain-processing measures, whereas self-reported physical functioning was linked to autonomic vagal modulation (RMSSD), and disability was more closely related to clinical pain intensity. Longitudinal analyses further support these domain-specific relationships. Improvements in objective lifting capacity were significantly associated with parallel changes in vagal autonomic regulation (ΔRMSSD), whereas improvements in subjective disability and physical functioning were more strongly linked to reductions in central sensitization symptoms and pain catastrophizing, respectively. Overall, the multivariate findings suggest a trend in which objective functional outcomes may be more closely related to autonomic and experimental pain-processing variables, whereas subjective disability outcomes appear to be more strongly related to psychosocial factors.

## 4. Discussion

This systematic review synthesized the available evidence on the relationships among autonomic physiological measures, psychosocial factors, experimental pain processing, and functional outcomes in adults with persistent spinal pain. Overall, the findings revealed heterogeneous but potentially domain-specific associations. Relationships between autonomic regulation and psychosocial variables were generally weak and inconsistent, with only isolated associations between nonlinear HRV indices and pain catastrophizing [36,39]. Similarly, evidence linking autonomic measures with experimental pain-processing outcomes remains conflicting, as some studies reported associations with PPTs and conditioned pain modulation (CPM), whereas others failed to confirm these relationships after correcting for multiple comparisons [32,38]. In contrast, psychosocial variables were more consistently associated with subjective pain and disability, whereas autonomic regulation and experimental pain-processing measures were more closely related to objective functional performance and physiological pain modulation. Together, these findings suggest that persistent spinal pain involves partially dissociable physiological and psychosocial mechanisms that differentially influence symptom perception and functional capacity.

Multivariable analyses further supported this interpretation by showing that psychosocial variables, including kinesiophobia (TSK), pain catastrophizing (PCS), and central sensitization-related symptoms, were more strongly associated with self-reported disability and physical functioning, whereas objective outcomes such as lifting capacity were primarily related to autonomic regulation and experimental pain-processing measures [20,24]. These findings may explain why improvements in physiological parameters do not necessarily translate into immediate reductions in perceived disability. Consequently, relying exclusively on self-reported questionnaires may overlook important aspects of patient status. Since current guideline-based treatments for CLBP, chronic neck pain, and WAD generally achieve only small-to-moderate effects [19,40,41], combining HRV, QST, and validated psychosocial assessments may provide a more comprehensive characterization of patients and facilitate phenotype-based rehabilitation strategies targeting predominant autonomic, nociceptive, or psychosocial profiles [39].

The limited associations identified between autonomic regulation and psychosocial factors may appear unexpected considering the well-established relationship between psychological distress and reduced HRV [34,42]. A possible explanation is offered by the neurovisceral integration model, which proposes that chronic pain disrupts prefrontal regulation of autonomic, emotional, and cognitive processes [43,44]. Under persistent homeostatic stress, autonomic regulation shifts towards increased sympathetic activity together with reduced parasympathetic modulation [45,46]. Moreover, the widespread use of short-term resting HRV recordings may have reduced the ability to detect dynamic autonomic–psychological interactions that become more evident during stress exposure or daily activities rather than under resting laboratory conditions [36,47,48,49].

### 4.1. Strengths and Limitations

#### 4.1.1. Study-Level Limitations

To our knowledge, this is the first systematic review to simultaneously examine the relationships among autonomic physiological measures, psychosocial factors, and pressure algometry within the same cohorts of adults with persistent spinal pain. The review followed PRISMA recommendations and applied the NIH Quality Assessment Tool, providing a transparent and reproducible methodology. Furthermore, separating HRV metrics into time-domain (RMSSD, SDNN) and frequency-domain (LF, HF, LF/HF) measures allowed a more detailed characterization of autonomic alterations across CLBP, chronic neck pain, and WAD. Nevertheless, only six studies fulfilled the eligibility criteria, and their overall methodological quality was generally rated as fair. Small sample sizes and the predominance of cross-sectional designs limited statistical power and prevented establishing causal or temporal relationships between autonomic dysfunction, psychological impairments, and altered pain processing.

#### 4.1.2. Measurement Heterogeneity and Biological Confounders

Considerable methodological heterogeneity prevented quantitative meta-analysis. The included studies differed substantially in HRV metrics, anatomical locations used for pressure pain thresholds (PPTs), conditioned pain modulation (CPM), mechanical detection thresholds (MDT), and psychosocial assessments. In addition, the PCS and TSK have recognized limitations, as catastrophizing is not consistently associated with experimental pain sensitivity and TSK should not be interpreted as a standalone clinical measure [50,51]. Another important limitation concerns resting cardiovascular measures such as HR and BP, which are strongly influenced by non-autonomic factors, including medication use, physical fitness, pain intensity, anxiety, respiratory patterns, and caffeine intake. Consequently, these variables should be interpreted as general physiological indicators of cardiovascular autonomic status rather than isolated markers of sympathetic or parasympathetic activity. Moreover, resting laboratory measurements may not adequately reflect autonomic adaptations occurring during real-life pain-related activities and stress exposure [52].

#### 4.1.3. Statistical Limitations

The interpretation of the available evidence is also limited by inconsistent statistical approaches across studies. While some investigations applied rigorous corrections for multiple comparisons, including Bonferroni or Holm adjustments, others reported only uncorrected exploratory analyses. Importantly, several statistically significant cross-domain associations became non-significant after appropriate correction procedures, suggesting that some reported findings may represent type I errors. Therefore, current evidence should be interpreted as preliminary and requires confirmation through adequately powered studies using standardized statistical methods.

#### 4.1.4. Review-Level Limitations

At the review level, the strict eligibility criteria requiring simultaneous assessment of autonomic, psychosocial, and pain-processing variables within the same cohort reduced the number of eligible studies. Although this approach minimized population-level bias and allowed true within-sample analyses, it substantially restricted the available evidence. Additionally, limiting the search to peer-reviewed articles published in English or Spanish may have introduced language bias by excluding relevant studies published in other languages.

### 4.2. Clinical Implications and Suggestions for Future Research

The findings support the use of multidimensional assessment in patients with persistent spinal pain, as subjective disability and pain intensity do not necessarily reflect objective physiological dysfunction. Future rehabilitation programs may benefit from combining objective autonomic markers, including portable HRV monitoring (RMSSD, LF/HF), quantitative sensory testing (PPT, CPM, and WUR), and validated psychosocial questionnaires such as the TSK, PCS, and CSI. Identifying whether autonomic, nociceptive, or psychosocial mechanisms predominate in individual patients may facilitate more personalized rehabilitation strategies. For example, patients with autonomic dysregulation and reduced PPTs may respond better to interventions targeting autonomic function, whereas individuals presenting greater psychosocial impairment may benefit from cognitive–behavioral approaches aimed at reducing kinesiophobia and pain catastrophizing. Future research should prioritize large prospective longitudinal studies that evaluate the temporal evolution of these domains from the acute stage to chronic spinal pain, including PSPS types 1 and 2. Standardized protocols for HRV acquisition, QST procedures, and statistical reporting are essential to facilitate future meta-analyses. In addition, future studies should investigate other psychosocial factors, including anxiety, anger, negative affect, and social rejection, as well as dynamic autonomic responses and baroreflex function during experimental pain rather than relying exclusively on resting cardiovascular measures [53].

## 5. Conclusions

In conclusion, this systematic review suggests that the autonomic, psychosocial, and pain-processing domains in persistent spinal pain populations manifest preliminary, domain-specific patterns of association rather than uniform pathophysiological mechanisms. A clear divergence emerged between subjective and objective dimensions: while subjective patient-reported outcomes, such as clinical disability perception, remain consistently linked to psychosocial factors, objective measures, including laboratory-based sensory testing and physical functional performance, exhibit closer relationships with experimental pain processing and proxies of parasympathetic modulation. Although multivariable models tentatively highlight specific psychosocial factors, such as fear of movement, as potential predictors of clinical pain exacerbation, these exploratory findings rest on limited primary datasets and must be interpreted with strict caution. Overall, the current evidence base remains preliminary, constrained by a small number of studies, variable measurement protocols, and predominantly cross-sectional designs. Consequently, future highly standardized longitudinal research utilizing rigorous statistical controls for multiple comparisons is warranted to validate these tentative cross-domain relationships and support specialized, research-informed clinical practices.

## Figures and Tables

**Table 1:** Summary and baseline characteristics of the included studies and their populations.

Study (Author, Year)	Bandeira et al., 2021 [39]	De Kooning et al., 2015 [38]	Santos-de-Araújo et al., 2019 [36]	Ansuategui Echeita et al., 2022 [37]	Kang et al., 2012 [20]	White et al., 2022 [32]
Study design	Case–control study	Case–control study	Cross-sectional study	Observational prospective cohort study	Cross-sectional study	Cross-sectional study
Location	Brazil	Belgium	Brazil	The Netherlands	Taiwan	Australia
Population	CLBP	Chronic WAD	Chronic neck pain	CLBP	Chronic neck pain	Chronic WAD
Sample size (n)	47	30	15	76	121	36
Pain duration	65 months	NR	36.40 (22.58) months	2.2 [1.3–4.2] years ^a^	NR	22 [36] months ^a^
Age (years), Mean (SD)	46.0 (9.60)	43.6 (9.44)	22.46 (3.09)	40.0 [31.3–50.8] ^a^	41.2 (13.9)	40.1 (14.6)
Female, n (%)	26 (55.3)	24 (80.0)	9 (60.0)	45 (59.2)	91 (75.2)	28 (78)
BMI (kg/m^2^), Mean (SD)	NR	NR	25.35 (4.87)	26.7 [24.2–30.6] ^a^	22.7 (3.5)	24.6 (5.0)
Pain severity (0–10 or vas 0–100), Mean (SD)	4.0 (1.01)	NR	NR	5.1 [2.7–6.6] ^a^	4.1 (2.8)	51 [32] ^a^
Kinesiophobia (TSK)	45.7 (6.79)	NR	34.66 (7.52)	NR	NR	37 (8)
PCS, Mean (SD)	27.5 (8.15)	17.17 (12.01)	1.06 (1.02)	18.0 [11.0–27.0] ^a^	NR	11 [14] ^a^
NDI, Mean (SD)	NR	44.27 (13.39)	8.80 (4.75)	NR	11.0 (8.5)	37 [20] ^a^
Conclusion	Patients with CLBP presented changes in sympathovagal balance during passive visualization of daily activities, along with higher pain sensitivity and pain intensity.	Dysfunctional endogenous pain inhibition (CPM) and lowered PPTs appear unrelated to autonomic responses to pain in WAD. No distinct dysfunction in the autonomic response to experimental pain was observed.	HRV indices were significantly associated with pain intensity, disability, and catastrophizing; worse linear and nonlinear HRV indices were correlated with conditions of more intense and disabling neck pain.	CS indicators were weakly related to functioning. Longitudinally, decreases in CS indicators (including RMSSD) were weakly to moderately related to increases in functioning (e.g., lifting capacity).	Reduced HRV was significantly associated with a higher degree of subjective disability, higher pain intensity, older age, poor sleep quality, and high psychological distress.	No significant correlations were found between resting HR or BP and pain processing or psychological variables in patients with chronic WAD. The expected association between blood pressure and pain sensitivity (hypertension-associated hypoalgesia) may be disrupted in chronic WAD.

^a^ Values are reported as median [Interquartile Range/IQR] rather than mean (SD). Pain severity scales: Bandeira et al. [39] and Kang et al. [20] used a 0–10 scale to assess pain severity. Ansuategui Echeita et al. [37] used a 0–10 VAS. White et al. [32] used a 0–100 mm VAS. Pain catastrophizing: Santos-de-Araújo et al. [36] used the Catastrophic Thoughts about Pain Scale (CTPS; 0–5) instead of the standard PCS. Missing Data: “NR” stands for Not Reported (or not applicable to the chronic pain group). Chronic Low Back Pain; Conditioned Pain Modulation; Heart Rate Variability; Neck Disability Index; Pain Catastrophizing Scale; Pressure Pain Threshold; Root Mean Square of Successive Differences; Tampa Scale of Kinesiophobia; Visual Analogue Scale; Whiplash-Associated Disorders.

## Data Availability

The original contributions presented in this study are included in the article/Supplementary Material. Further inquiries can be directed to the corresponding author.

## Supplementary Materials

The following supporting information can be downloaded at: https://www.mdpi.com/article/10.3390/jcm15145571/s1. PRISMA 2020 Checklist; Table S1: Detailed search strategy for each database; Table S2: Quality assessment using NIH tool for observational cohort and cross-sectional studies; Table S3: Quality assessment using NIH tool for case-control studies; Table S4: Bivariate associations between autonomic and psychosocial variables in individuals with persistent spinal pain; Table S5: Bivariate associations between autonomic variables and pain processing measures in individuals with persistent spinal pain; Table S6. Bivariate associations between psychosocial variables and pain processing or functional outcomes in individuals with persistent spinal pain; Table S7: Cross-domain associations between autonomic variables, pain measures, and disability outcomes in individuals with persistent spinal pain; Table S8: Multivariable regression analyses examining associations across autonomic, psychosocial, and pain processing domains in individuals with persistent spinal pain.

## Abbreviations

The following abbreviations are used in this manuscript.

CLBP	Chronic Low Back Pain
WAD	Whiplash-Associated Disorder
PPT	Pressure Pain Threshold
HRV	Heart Rate Variability
BP	Blood Pressure
HR	Heart Rate
QST	Quantitative Sensory Testing
PRISMA	Preferred Reporting Items for Systematic Reviews and Meta-Analyses
MeSH	Medical Subject Headings
TSK	Tampa Scale for Kinesiophobia
PCS	Pain Catastrophizing Scale
NDI	Neck Disability Index
LF	Low Frequency
HF	High Frequency
LF/HF	Low Frequency/High Frequency Ratio
LF nu	Low Frequency (normalized units)
HF nu	High Frequency (normalized units)
RMSSD	Root Mean Square of Successive Differences
SDNN	Standard Deviation of Normal-to-Normal Intervals
TINN	Triangular Interpolation of NN Interval Histogram
Mean RR	Mean R-R Interval
STD-RR	Standard Deviation of R-R Intervals
RR Tri	RR Triangular Index
SD1	Standard Deviation 1 (Poincaré plot)
SD2	Standard Deviation 2 (Poincaré plot)
SD2/SD1	Ratio of SD2 to SD1
Alpha 1	Short-term fractal scaling exponent
Alpha 2	Long-term fractal scaling exponent
ApEn	Approximate Entropy
SampEn	Sample Entropy
SBP	Systolic Blood Pressure
DBP	Diastolic Blood Pressure
CPM	Conditioned Pain Modulation
WUR	Wind-Up Ratio
MPT	Mechanical Pain Threshold
MDT	Mechanical Detection Threshold
HADS	Hospital Anxiety and Depression Scale
HADS-A	Hospital Anxiety and Depression Scale—Anxiety Subscale
HADS-D	Hospital Anxiety and Depression Scale—Depression Subscale
IES	Impact of Event Scale
PCL-S	Post-Traumatic Stress Disorder Checklist—Stressor Specific
CTPS	Catastrophic Thoughts about Pain Scale
CHQ-12	Chinese Health Questionnaire-12
VAS	Visual Analogue Scale
NRS	Numerical Rating Scale
NRSm	Numerical Rating Scale (movement)
PHODA	Photograph Series of Daily Activities
PDI	Pain Disability Index
CSI-A	Central Sensitization Inventory—Part A
Rand36-PF	RAND 36-Item Health Survey—Physical Functioning
WAS	Work Ability Score
NIH	National Institutes of Health
FDR	False Discovery Rate
NN50	Number of NN interval differences > 50 ms
pNN50	Percentage of NN50
TP	Total Power
Δ (Delta)	Change/Difference
NR	Not Reported
rpartial	Partial Correlation Coefficient
