# Supplementary material for: Autonomic Dysfunction, Psychosocial Factors, and Pain Sensitivity in Persistent Spinal Pain: A Systematic Review
Source: J Clin Med. 2026 Jul 16;15(14):5571. doi: 10.3390/jcm15145571 (PMC13413165; doi:10.3390/jcm15145571)
Supplement: Supplementary file 1 [file jcm-15-05571-s001.zip › jcm-4388986-supplementary Tables.pdf]

Supplementary Tables

Supplementary Table S1. Detailed search strategy for each database. 2

Supplementary Table S2. Quality assessment using NIH tool for observational cohort and cross-sectional studies. 7

Supplementary Table S3. Quality assessment using NIH tool for case-control studies. 8

Supplementary Table S4. Bivariate associations between autonomic and psychosocial variables in individuals with persistent spinal pain. 9

Supplementary Table S5. Bivariate associations between autonomic variables and pain processing measures in individuals with persistent spinal pain. 11

Supplementary Table S6. Bivariate associations between psychosocial variables and pain processing or functional outcomes in individuals with persistent spinal pain. 12

Supplementary Table S7. Cross-domain associations between autonomic variables, pain measures, and disability outcomes in individuals with persistent spinal pain. 13

Supplementary Table S8. Multivariable regression analyses examining associations across autonomic, psychosocial, and pain processing domains in individuals with persistent spinal pain. 16

Supplementary Table S1. Detailed search strategy for each database.

Database	Search term	Filter	Results
**PubMed**	("Low Back Pain"[Mesh] OR "Back Pain"[Mesh] OR "Neck Pain"[Mesh] OR "Whiplash Injuries"[Mesh] OR "chronic low back pain" OR "chronic low-back pain" OR "chronic lumbar pain" OR "chronic lumbar back pain" OR "chronic lumbago" OR "chronic back pain" OR "persistent low back pain" OR "persistent back pain" OR "chronic neck pain" OR "chronic cervical pain" OR "persistent neck pain" OR "persistent cervical pain" OR "whiplash associated disorder" OR "whiplash-associated disorder" OR whiplash OR "chronic whiplash" OR "persistent spinal pain" OR "persistent spinal pain syndrome" OR "persistent spinal pain syndrome type 2" OR "failed back surgery syndrome" OR "post spinal surgery syndrome" OR "post-laminectomy syndrome" OR "post laminectomy syndrome" OR "chronic spinal pain") AND ("Autonomic Nervous System"[Mesh] OR "Heart Rate"[Mesh] OR "Heart Rate Variability"[Mesh] OR "Blood Pressure"[Mesh] OR "Electrodermal Activity"[Mesh] OR autonomic OR "autonomic nervous system" OR sympathetic OR parasympathetic OR "vagal tone" OR cardiovascular OR "cardiovascular regulation" OR "heart rate variability" OR "respiratory sinus arrhythmia" OR "root mean square of successive differences" OR "low frequency high frequency ratio" OR "resting heart rate" OR "heart rate" OR "resting blood pressure" OR "blood pressure" OR "systolic blood pressure" OR "diastolic blood pressure" OR electrodermal OR "electrodermal activity" OR "skin conductance" OR "skin conductance response" OR "skin conductance level" OR "sympathetic skin response" OR "autonomic function" OR "autonomic regulation") AND ("Psychological Factors"[Mesh] OR "Adaptation, Psychological"[Mesh] OR "Anxiety"[Mesh] OR "Depression"[Mesh] OR psychological OR psychosocial OR distress OR "psychological distress" OR emotion OR affect OR coping OR "pain catastrophizing" OR "pain catastrophizing scale" OR "catastrophic thinking"	All Filed	**102**

	OR catastrophizing OR kinesiophobia OR "fear of movement" OR "tampa scale of kinesiophobia" OR "fear avoidance" OR "fear avoidance beliefs" OR "fear avoidance beliefs questionnaire" OR "central sensitization" OR "central sensitization syndrome" OR "central sensitization inventory" OR depression OR "depressive symptoms" OR anxiety OR "anxiety symptoms" OR "psychological factors" OR "psychosocial factors") AND ("Pain Measurement"[Mesh] OR "Pain Threshold"[Mesh] OR "Quantitative Sensory Testing"[Mesh] OR "pressure pain threshold" OR "pressure pain thresholds" OR "pressure algometry" OR algometry OR algometer OR "pressure algometer" OR "mechanical pain threshold" OR "mechanical pain sensitivity" OR "quantitative sensory testing" OR "experimental pain testing" OR "experimental pain" OR "pain sensitivity" OR "pain threshold" OR "pain processing" OR "pain modulation" OR "conditioned pain modulation" OR "temporal summation" OR "mechanical nociceptive threshold")		
**Scopus**	("chronic low back pain" OR "chronic low-back pain" OR "chronic lumbar pain" OR "chronic lumbar back pain" OR "chronic lumbago" OR "chronic back pain" OR "persistent low back pain" OR "persistent back pain" OR "chronic neck pain" OR "chronic cervical pain" OR "persistent neck pain" OR "persistent cervical pain" OR "whiplash associated disorder" OR "whiplash-associated disorder" OR "whiplash" OR "chronic whiplash" OR "persistent spinal pain" OR "persistent spinal pain syndrome" OR "persistent spinal pain syndrome type 2" OR "failed back surgery syndrome" OR "post spinal surgery syndrome" OR "post-laminectomy syndrome" OR "post laminectomy syndrome" OR "chronic spinal pain") AND ("autonomic nervous system" OR "autonomic" OR "sympathetic" OR "parasympathetic" OR "vagal tone" OR "cardiovascular" OR "cardiovascular regulation" OR "heart rate variability" OR "respiratory sinus arrhythmia" OR "root mean square of successive differences" OR "low frequency high frequency ratio" OR "resting heart rate" OR "heart rate" OR "resting blood pressure" OR "blood pressure" OR "systolic blood pressure" OR "diastolic blood pressure" OR "electrodermal" OR "electrodermal activity" OR "skin conductance" OR "skin conductance response" OR "skin conductance level" OR "sympathetic skin response" OR "autonomic function" OR "autonomic regulation") AND	Article title, abstract, keywords	**45**

	("psychological" OR "psychosocial" OR "psychological distress" OR "distress" OR "emotion" OR "affect" OR "coping" OR "pain catastrophizing" OR "pain catastrophizing scale" OR "catastrophic thinking" OR "catastrophizing" OR "kinesiophobia" OR "fear of movement" OR "tampa scale of kinesiophobia" OR "fear avoidance" OR "fear avoidance beliefs" OR "fear avoidance beliefs questionnaire" OR "central sensitization" OR "central sensitization syndrome" OR "central sensitization inventory" OR "depression" OR "depressive symptoms" OR "anxiety" OR "anxiety symptoms" OR "psychological factors" OR "psychosocial factors") AND ("pressure pain threshold" OR "pressure pain thresholds" OR "pressure algometry" OR "algometry" OR "algometer" OR "pressure algometer" OR "mechanical pain threshold" OR "mechanical pain sensitivity" OR "quantitative sensory testing" OR "experimental pain testing" OR "experimental pain" OR "pain sensitivity" OR "pain threshold" OR "pain processing" OR "pain modulation" OR "conditioned pain modulation" OR "temporal summation" OR "mechanical nociceptive threshold")		
**Web of Science**	("chronic low back pain" OR "chronic low-back pain" OR "chronic lumbar pain" OR "chronic lumbar back pain" OR "chronic lumbago" OR "chronic back pain" OR "persistent low back pain" OR "persistent back pain" OR "chronic neck pain" OR "chronic cervical pain" OR "persistent neck pain" OR "persistent cervical pain" OR "whiplash associated disorder" OR "whiplash-associated disorder" OR "whiplash" OR "chronic whiplash" OR "persistent spinal pain" OR "persistent spinal pain syndrome" OR "persistent spinal pain syndrome type 2" OR "failed back surgery syndrome" OR "post spinal surgery syndrome" OR "post-laminectomy syndrome" OR "post laminectomy syndrome" OR "chronic spinal pain")<br>**AND**<br>("autonomic nervous system" OR "autonomic regulation" OR "autonomic function" OR "sympathetic nervous system" OR "parasympathetic nervous system" OR "vagal tone" OR "cardiovascular regulation" OR "heart rate variability" OR "respiratory sinus arrhythmia" OR "root mean square of successive differences" OR "low frequency high frequency ratio"	All Filed	**31**

	OR "resting heart rate" OR "heart rate" OR "resting blood pressure" OR "blood pressure" OR "systolic blood pressure" OR "diastolic blood pressure" OR "electrodermal activity" OR "skin conductance" OR "skin conductance response" OR "skin conductance level" OR "sympathetic skin response")<br>**AND**<br>("psychological factors" OR "psychosocial factors" OR "psychological distress" OR "distress" OR "emotion" OR "affect" OR "coping" OR "pain catastrophizing" OR "pain catastrophizing scale" OR "catastrophic thinking" OR "catastrophizing" OR "kinesiophobia" OR "fear of movement" OR "tampa scale of kinesiophobia" OR "fear avoidance" OR "fear avoidance beliefs" OR "fear avoidance beliefs questionnaire" OR "central sensitization" OR "central sensitization syndrome" OR "central sensitization inventory" OR "depression" OR "depressive symptoms" OR "anxiety" OR "anxiety symptoms")<br>**AND**<br>("pressure pain threshold" OR "pressure pain thresholds" OR "pressure algometry" OR "algometry" OR "algometer" OR "pressure algometer" OR "mechanical pain threshold" OR "mechanical pain sensitivity" OR "quantitative sensory testing" OR "experimental pain testing" OR "experimental pain" OR "pain sensitivity" OR "pain threshold" OR "pain processing" OR "pain modulation" OR "conditioned pain modulation" OR "temporal summation" OR "mechanical nociceptive threshold")		
**Cochrane library**	("chronic low back pain" OR "chronic low-back pain" OR "chronic lumbar pain" OR "chronic lumbar back pain" OR "chronic lumbago" OR "chronic back pain" OR "persistent low back pain" OR "persistent back pain" OR "chronic neck pain" OR "chronic cervical pain" OR "persistent neck pain" OR "persistent cervical pain" OR "whiplash associated disorder" OR "whiplash-associated disorder" OR "whiplash" OR "chronic whiplash" OR "persistent spinal pain" OR "persistent spinal pain syndrome" OR "persistent spinal pain syndrome type 2" OR "failed back surgery syndrome" OR "post spinal surgery syndrome" OR "post-laminectomy syndrome" OR "post laminectomy syndrome" OR "chronic spinal	All Filed	**33**

	pain") AND ("autonomic nervous system" OR "autonomic" OR "sympathetic" OR "parasympathetic" OR "vagal tone" OR "cardiovascular" OR "cardiovascular regulation" OR "heart rate variability" OR "respiratory sinus arrhythmia" OR "root mean square of successive differences" OR "low frequency high frequency ratio" OR "resting heart rate" OR "heart rate" OR "resting blood pressure" OR "blood pressure" OR "systolic blood pressure" OR "diastolic blood pressure" OR "electrodermal" OR "electrodermal activity" OR "skin conductance" OR "skin conductance response" OR "skin conductance level" OR "sympathetic skin response" OR "autonomic function" OR "autonomic regulation") AND ("psychological" OR "psychosocial" OR "psychological distress" OR "distress" OR "emotion" OR "affect" OR "coping" OR "pain catastrophizing" OR "pain catastrophizing scale" OR "catastrophic thinking" OR "catastrophizing" OR "kinesiophobia" OR "fear of movement" OR "tampa scale of kinesiophobia" OR "fear avoidance" OR "fear avoidance beliefs" OR "fear avoidance beliefs questionnaire" OR "central sensitization" OR "central sensitization syndrome" OR "central sensitization inventory" OR "depression" OR "depressive symptoms" OR "anxiety" OR "anxiety symptoms" OR "psychological factors" OR "psychosocial factors") AND ("pressure pain threshold" OR "pressure pain thresholds" OR "pressure algometry" OR "algometry" OR "algometer" OR "pressure algometer" OR "mechanical pain threshold" OR "mechanical pain sensitivity" OR "quantitative sensory testing" OR "experimental pain testing" OR "experimental pain" OR "pain sensitivity" OR "pain threshold" OR "pain processing" OR "pain modulation" OR "conditioned pain modulation" OR "temporal summation" OR "mechanical nociceptive threshold")		

Supplementary Table S2. Quality assessment using NIH tool for observational cohort and cross-sectional studies.

Criteria	**Santos-De-Araújo et al., 2019 [36]**	**Ansuategui Echeita et al., 2022 [37]**	**Kang et al., 2012 [20]**	**White et al., 2022 [32]**
Study design	Cross-Sectional	Prospective Cohort	Cross-Sectional	Cross-Sectional
1. Research question stated?	Y	Y	Y	Y
2. Study population defined?	Y	Y	Y	Y
3. Participation rate at least 50%?	NR	NR	NR	NR
4. Same populations & uniform criteria?	Y	Y	Y	Y
5. Sample size justification?	Y	Y	N	Y
6. Exposure measured prior to outcome?	N	Y	N	N
7. Sufficient timeframe?	N	Y	N	N
8. Different levels of exposure?	Y	Y	Y	Y
9. Exposure measures clearly defined?	Y	Y	Y	Y
10. Exposure assessed more than once over time?	NA	Y	NA	NA
11. Outcome measures clearly defined?	Y	Y	Y	Y
12. Outcome assessors blinded?	Y	NR	NR	N
13. Loss to follow-up 20% or less?	NA	N *(26.3% drop-out)*	NA	NA
14. Confounding variables adjusted?	N	Y	Y	Y
**Overall rating**	**Fair**	**Good**	**Fair**	**Fair**

Y = Yes; **N** = No; **NR** = Not Reported; **NA** = Not Applicable (for cross-sectional studies, items related to follow-up timeframes, repeated exposures, and loss to follow-up are inherently not applicable and do not negatively affect the overall quality rating, in accordance with NIH guidelines).

Overall rating: Good, Fair, or Poor.

Supplementary Table S3. Quality assessment using NIH tool for case-control studies.

Criteria	Bandeira et al., 2021 [39]	De Kooning et al., 2015 [38]
1. Research question stated?	Y	Y
2. Study population defined?	Y	Y
3. Sample size justification?	Y	N
4. Controls from same/similar population?	Y	Y
5. Uniform inclusion/exclusion criteria?	Y	Y
6. Cases clearly defined/differentiated?	Y	Y
7. Random selection of controls?	NR	NR
8. Concurrent controls?	Y	Y
9. Exposure prior to outcome? *	NA	NA
10. Exposure measures clearly defined?	Y	Y
11. Assessors blinded?	N	NR
12. Confounding variables adjusted?	Y	Y
**Overall rating**	**Fair**	**Fair**

Y = Yes; **N** = No; **NR** = Not Reported; **NA** = Not Applicable. **Overall rating:** Good, Fair, or Poor. *: (Q9): In experimental pain studies using physiological markers, the exposure (e.g., HRV/autonomic response) and the outcome (e.g., pain sensitivity or PPT) are measured concurrently within the same session. Therefore, assessing whether the exposure preceded the outcome is generally not applicable for these designs.

Supplementary Table S4. Bivariate associations between autonomic and psychosocial variables in individuals with persistent spinal pain.

Study (Year)	Design	N	Population	Autonomic Variable	Psychosocial Variable	Analysis	r	p-value	Significance	Notes
**Bandeira et al., 2021 [39]**	Case-control	47	CLBP	HRV freq-domain: ΔLF, ΔHF, ΔLF/HF	Kinesiophobia (TSK)	Pearson	NR	≥ 0.05	Non-significant	Δ = post-task minus pre-task; LF, HF, and LF/HF showed identical null result
				HRV freq-domain: ΔLF, ΔHF, ΔLF/HF	Catastrophizing (PCS)	Pearson	NR	≥ 0.05	Non-significant	All three freq-domain indices showed identical null result
				HRV freq-domain: ΔLF, ΔHF, ΔLF/HF	Anxiety (HADS-A)	Pearson	NR	≥ 0.05	Non-significant	
				HRV freq-domain: ΔLF, ΔHF, ΔLF/HF	Depression (HADS-D)	Pearson	NR	≥ 0.05	Non-significant	
**De Kooning et al., 2015 [38]**	Case-control	30	Chronic WAD	HRV time-domain: SDNN, RMSSD	Neck Disability Index (NDI)	Pearson (sex-corrected)	NR	> 0.01	Non-significant	significance threshold p < 0.01
				HRV freq-domain: LF, HF, LF/HF; HR; skin conductance	Neck Disability Index (NDI)	Pearson (sex-corrected)	NR	> 0.01	Non-significant	
				HRV time-domain: SDNN, RMSSD	Posttraumatic Stress Reaction (IES)	Pearson (sex-corrected)	NR	> 0.01	Non-significant	
				HRV freq-domain: LF, HF, LF/HF; HR; skin conductance	Posttraumatic Stress Reaction (IES)	Pearson (sex-corrected)	NR	> 0.01	Non-significant	
**Santos-de-Araújo et al., 2019 [36]**	Cross-sectional	15	Chronic neck pain	HRV time-domain: Mean RR, RMSSD, STD-RR, RR Tri, TINN	Kinesiophobia (TSK)	Pearson	NR	≥ 0.05	Non-significant	Consistent across supine, sitting, and standing positions
				HRV freq-domain: LF (nu), HF (nu), LF/HF	Kinesiophobia (TSK)	Pearson	NR	≥ 0.05	Non-significant	Consistent across all positions
				HRV nonlinear: SD1, SD2, SD2/SD1, Alpha 1, Alpha 2, ApEn, SampEn	Kinesiophobia (TSK)	Pearson	NR	≥ 0.05	Non-significant	Consistent across all positions
				HRV nonlinear: SD2/SD1 ratio (supine)	Catastrophizing (CTPS)	Pearson	0.384	< 0.05	Significant	Only significant autonomic–psychosocial correlation in the study; supine position only
				HRV time-domain: RMSSD, STD-RR, Mean RR, RR Tri	Catastrophizing (CTPS)	Pearson	-0.211 to -0.346	≥ 0.05	Non-significant	Supine position; approaching but not reaching significance
				HRV freq-domain: LF (nu), HF (nu), LF/HF	Catastrophizing (CTPS)	Pearson	-0.203 to 0.349	≥ 0.05	Non-significant	Supine position; LF/HF r = 0.349 approached significance

Study (Year)	Design	N	Population	Autonomic Variable	Psychosocial Variable	Analysis	r	p-value	Significance	Notes
**White et al., 2022 [32]**	Cross-sectional	36	Chronic WAD	HR, SBP, DBP (resting)	Pain Catastrophizing Scale (PCS)	Spearman	-0.16 to 0.14	0.34–0.99	Non-significant (FDR corrected)	Benjamini-Hochberg FDR 5% applied
				HR, SBP, DBP (resting)	Kinesiophobia (TSK)	Spearman/Pearson	-0.18 to 0.04	0.29–0.84	Non-significant (FDR corrected)	
				HR, SBP, DBP (resting)	Posttraumatic Stress Symptoms (PCL-S)	Spearman	-0.14 to -0.03	0.42–0.87	Non-significant (FDR corrected)	

Bivariate correlations between autonomic and psychosocial variables in individuals with persistent spinal pain. **r** denotes the Pearson or Spearman correlation coefficient as specified per row. All analyses are bivariate unless otherwise stated. In De Kooning et al. [38], correlations were conducted within each group and adjusted for sex, with a significance threshold of $p < 0.01$. In White et al. [32], a Benjamini–Hochberg false discovery rate (FDR) correction at 5% was applied. Frequency-domain HRV indices (LF, HF, LF/HF) in Bandeira et al. [39] are reported as change scores (Δ), calculated as post-task minus pre-task. Abbreviations: **HRV**: Heart rate variability; **LF**: Low-frequency power (0.04–0.15 Hz); **HF**: High-frequency power (0.15–0.40 Hz); **LF/HF**: Low-frequency to high-frequency ratio; **SDNN**: Standard deviation of normal-to-normal RR intervals; **RMSSD**: Root mean square of successive differences; **HR**: Heart rate; **SBP**: Systolic blood pressure; **DBP**: Diastolic blood pressure; **TSK**: Tampa Scale for Kinesiophobia; **PCS**: Pain Catastrophizing Scale; **HADS-A**: Hospital Anxiety and Depression Scale—Anxiety; **HADS-D**: Hospital Anxiety and Depression Scale—Depression; **NDI**: Neck Disability Index; **IES**: Impact of Event Scale; **PCL-S**: PTSD Checklist—Civilian Version; **CLBP**: Chronic low back pain; **WAD**: Whiplash-associated disorder; **FDR**: False discovery rate; **NR**: Not reported; **Δ**: Change score (post − pre).

Supplementary Table S5. Bivariate associations between autonomic variables and pain processing measures in individuals with persistent spinal pain.

Study (Year)	Design	N	Population	Autonomic Variable	Pain Processing Variable	Analysis	r	p-value	Significance	Notes
**De Kooning et al., 2015 [38]**	Case-control	30	Chronic WAD	HR (resting)	PPT — upper trapezius	Pearson (sex-corrected)	0.480	0.008	Significant	Higher resting HR associated with higher PPT (reduced mechanical sensitivity)
				HRV freq-domain: LF (resting)	CPM effect — upper trapezius	Pearson (sex-corrected)	-0.473	0.01	Significant	a larger CPM effect at the upper trapezius was associated with lower LEG at rest
				HRV time-domain: SDNN reactivity (during pain − rest)	CPM effect — upper trapezius	Pearson (sex-corrected)	0.494	0.006	Significant	SDNN reactivity = change in SDNN from rest to cuff inflation; greater reactivity = better CPM
**White et al., 2022 [32]**	Cross-sectional	36	Chronic WAD	HR (resting)	PPT (cervical spine, hand, tibialis anterior)	Spearman/Pearson	-0.07 to -0.09	0.62–0.70	Non-significant (FDR corrected)	All three PPT sites showed same null result; FDR 5% applied
				SBP (resting)	PPT (cervical spine, hand, tibialis anterior)	Spearman/Pearson	0.12 to 0.27	0.11–0.51	Non-significant (FDR corrected)	All three PPT sites are non-significant
				DBP (resting)	PPT (cervical spine, hand, tibialis anterior)	Spearman/Pearson	0.05 to 0.16	0.34 to 0.78	Non-significant (FDR corrected)	All three PPT sites are non-significant
				HR (resting)	Temporal summation — cervical spine (WUR)	Spearman	-0.01	0.98	Non-significant (FDR corrected)	
				HR (resting)	Temporal summation — hand (WUR)	Spearman	0.41	0.02	Non-significant (FDR corrected)	Approached significance before correction; did not survive FDR 5%
				SBP, DBP (resting)	Temporal summation — cervical spine & hand (WUR)	Spearman	-0.05 to 0.14	0.42 to 0.77	Non-significant (FDR corrected)	
				HR, SBP, DBP (resting)	CPM (kPa and % baseline)	Spearman	-0.10 to 0.12	0.42 to 0.99	Non-significant (FDR corrected)	All combinations non-significant

Bivariate correlations between autonomic variables and pain processing measures (pressure pain threshold, conditioned pain modulation, and temporal summation) in individuals with persistent spinal pain. **r** denotes the Pearson or Spearman correlation coefficient as specified per row. In De Kooning et al. [38], analyses were performed within the chronic WAD group and adjusted for sex ($p < 0.01$). In White et al. [32], a Benjamini–Hochberg FDR correction at 5% was applied. Multiple PPT sites (cervical spine, hand, tibialis anterior) are grouped when results were consistent across sites. Abbreviations: **HRV**: Heart rate variability; **LF**: Low-frequency power; **HF**: High-frequency power; **SDNN**: Standard deviation of normal-to-normal RR intervals; **RMSSD**: Root mean square of successive differences; **HR**: Heart rate; **SBP**: Systolic blood pressure; **DBP**: Diastolic blood pressure; **PPT**: Pressure pain threshold; **CPM**: Conditioned pain modulation; **WUR**: Wind-up ratio; **CLBP**: Chronic low back pain; **WAD**: Whiplash-associated disorder; **FDR**: False discovery rate; **NR**: Not reported.

Supplementary Table S6. Bivariate associations between psychosocial variables and pain processing or functional outcomes in individuals with persistent spinal pain.

Study (Year)	Design	N	Population	Psychosocial Variable	Pain Processing / Functional Variable	Analysis	r	p-value	Significance	Notes
**Bandeira et al., 2021 [39]**	Case-control	47	CLBP	Kinesiophobia (TSK)	Delta pain intensity (pre/post PHODA task)	Pearson	0.38	0.009	Significant	Only significant psychosocial–pain association in this study
				Catastrophizing (PCS); Anxiety (HADS-A); Depression (HADS-D)	Delta pain intensity (pre/post PHODA task)	Pearson	NR	≥ 0.05	Non-significant	All three variables showed identical null result
**Ansuategui Echeita et al., 2022 [37]**	Prospective cohort (cross-sectional)	76	CLBP	CSI-A (central sensitization symptoms)	Lifting capacity, physical functioning (Rand36-PF), disability (PDI), work ability (WAS)	Spearman partial (age/sex corrected)	partial = −0.11 to 0.15	≥ 0.01	Non-significant	All four functioning outcomes weak and non-significant in bivariate analysis; see Table 5 for adjusted regression results
	Prospective cohort (longitudinal)	≤56		ΔCSI-A (change in CS symptoms)	ΔDisability (PDI)	Pearson/Spearman	0.44	< 0.01	Significant	Moderate association: reduction in CS symptoms linked to reduction in disability
		≤56		ΔCSI-A (change in CS symptoms)	ΔLifting capacity; ΔPhysical functioning (Rand36-PF); ΔWork ability (WAS)	Pearson/Spearman	partial = -0.37 to 0.32	≥ 0.01	Non-significant	ΔCSI-A not significantly associated with any other functional outcome longitudinally

Bivariate correlations between psychosocial variables and pain processing or functional outcomes in individuals with persistent spinal pain. **r** denotes the Pearson or Spearman correlation coefficient as specified per row. In Ansuategui Echeita et al. [37], the significance threshold for bivariate analyses was set at $p < 0.01$ to account for multiple comparisons. Cross-sectional analyses used baseline data, whereas longitudinal analyses used change scores (Δ). Abbreviations: **TSK**: Tampa Scale for Kinesiophobia; **PCS**: Pain Catastrophizing Scale; **HADS-A**: Hospital Anxiety and Depression Scale—Anxiety; **HADS-D**: Hospital Anxiety and Depression Scale—Depression; **CSI-A**: Central Sensitization Inventory (Part A); **ΔCSI-A**: Change in CSI-A score from baseline to discharge; **PHODA**: Photograph Series of Daily Activities; **PDI**: Pain Disability Index; **Rand36-PF**: RAND-36 Physical Functioning subscale; **WAS**: Work Ability Score; **CLBP**: Chronic low back pain; **NR**: Not reported; **Δ**: Change score.

Supplementary Table S7. Cross-domain associations between autonomic variables, pain measures, and disability outcomes in individuals with persistent spinal pain.

Study (Year)	Design	N	Population	Variable 1	Variable 2	Domain Pairing	Analysis	r	p-value	Significance	Notes
**Kang et al., 2012 [20]**	Cross-sectional	121	Chronic neck pain	HRV time-domain: SDNN, RMSSD, TINN, NN50, pNN50	Disability (NDI)	Autonomic vs Disability	Pearson	−0.322 to −0.410	0.001	Significant	All five time-domain indices significantly and negatively associated with disability
				HRV freq-domain: LF power, HF power, Total Power	Disability (NDI)	Autonomic vs Disability	Pearson	−0.211 to −0.354	(LF)= 0.011, (HF)= 0.02, (TP)= <0.001	Significant	LF: r = −0.230, p = 0.011; HF: r = −0.211, p = 0.02; TP: r = −0.354, p < 0.001
				HRV freq-domain: LF/HF ratio	Disability (NDI)	Autonomic vs Disability	Pearson	−0.033	0.718	Non-significant	LF/HF ratio non-significant unlike absolute LF and HF values
				Mean HR	Disability (NDI)	Autonomic vs Disability	Pearson	-0.114	0.213	Non-significant	
				Pain intensity (VAS)	Disability (NDI)	Pain vs Disability	Pearson	0.691	< 0.001	Significant	Strongest bivariate association in this study
				PPT	Disability (NDI)	Pain Processing vs Disability	Pearson	−0.166	0.069	Non-significant	
				Psychological distress (CHQ-12)	Disability (NDI)	Psychosocial vs Disability	Pearson	0.636	< 0.001	Significant	CHQ-12 used here as a validated measure of psychological distress
**Ansuategui Echeita et al., 2022 [37]**	cross-sectional	76	CLBP	HRV time-domain: RMSSD	Physical functioning (Rand36-PF)	Autonomic vs Function	Spearman partial	NR	≥ 0.01	Non-significant (bivariate)	Significant only in adjusted regression
				HRV time-domain: RMSSD	Lifting capacity; disability (PDI); work ability (WAS)	Autonomic vs Function	Spearman partial	NR	≥ 0.01	Non-significant	RMSSD not significantly associated with other functional outcomes in bivariate analysis
		≤56		HRV time-domain: ΔRMSSD	ΔLifting capacity	Autonomic vs Function (longitudinal)	Pearson/Spearman	0.41	< 0.01	Significant	Moderate association: improvement in vagal tone linked to improvement in lifting capacity
**White et al., 2022 [32]**	Cross-sectional	36		HR, SBP, DBP (resting)	Pain intensity (VAS)	Autonomic vs Pain	Spearman	-0.25 to 0.30	0.08–0.14	Non-significant (FDR corrected)	Trend only; did not survive FDR 5% correction
				HR, SBP, DBP (resting)	Disability (NDI)	Autonomic vs Disability	Pearson	-0.19 to 0.03	0.27–0.87	Non-significant (FDR corrected)	
				HR, SBP, DBP (resting)	Pain duration (months)	Autonomic vs Pain Duration	Spearman	-0.12 to 0.06	0.47–0.88	Non-significant (FDR corrected)	

Study (Year)	Design	N	Population	Variable 1	Variable 2	Domain Pairing	Analysis	r	p-value	Significance	Notes
**De Kooning et al., 2015 [38]**	Case-control	30	Chronic WAD	HR (resting)	PPT — quadriceps	Autonomic vs Pain Processing	Pearson (sex-corrected)	NR	> 0.01	Non-significant	HR vs quadriceps PPT non-significant; contrast with significant HR vs trapezius PPT (Table 2)
**Santos-de-Araújo et al., 2019 [36]**	Cross-sectional	30	Chronic neck pain	HRV time-domain: Mean RR intervals	Pain during movement (NRSm)	Autonomic vs Pain	Pearson	-0.411 to -0.581	< 0.05	Significant	Consistent across supine, sitting, and standing positions
				Mean HR	Pain during movement (NRSm)	Autonomic vs Pain	Pearson	0.432 to 0.603	< 0.05	Significant	Consistent across supine, sitting, and standing positions
				HRV time-domain: STD-RR, RR Tri; HRV nonlinear: SD2	Pain during movement (NRSm)	Autonomic vs Pain	Pearson	-0.382 to -0.477	< 0.05	Significant	Supine position only
				HRV freq-domain: LF (nu) — standing	Pain at rest (NRSr)	Autonomic vs Pain	Pearson	-0.394	< 0.05	Significant	Standing position only; higher LF (sympathetic index) = lower resting pain
				HRV freq-domain: HF (nu) — standing	Pain at rest (NRSr)	Autonomic vs Pain	Pearson	0.388	< 0.05	Significant	Standing position only; inverse of LF finding
				HRV time-domain: Mean RR intervals	Disability (NDI)	Autonomic vs Disability	Pearson	-0.368 to -0.421	< 0.05	Significant	Consistent across supine and sitting positions
				Mean HR	Disability (NDI)	Autonomic vs Disability	Pearson	0.372 to 0.426	< 0.05	Significant	Consistent across supine and sitting positions
				HRV time-domain: RMSSD, STD-RR, RR Tri; HRV nonlinear: SD1, SD2	Disability (NDI)	Autonomic vs Disability	Pearson	-0.362 to -0.391	< 0.05	Significant	Supine position only; all five indices significantly and negatively associated with NDI
				HRV time-domain: Mean RR; Mean HR	Pain chronicity (months)	Autonomic vs Pain Duration	Pearson	-0.445 (RR); r = 0.431 (HR)	< 0.05	Significant	Supine position; opposite signs reflect same physiological phenomenon

Bivariate cross-domain correlations between autonomic variables, pain measures, and disability or functional outcomes in individuals with persistent spinal pain. Only chronic pain populations are included. **r** denotes the Pearson or Spearman correlation coefficient as specified per row. In Kang et al. [20], frequency-domain HRV indices are presented separately due to differences in statistical significance across components. In White et al. [32], a Benjamini–Hochberg FDR correction at 5% was applied. In Ansuategui Echeita et al. [37], some associations were non-significant in bivariate analyses but significant in adjusted regression models. Abbreviations: **HRV**: Heart rate variability; **LF**: Low-frequency power; **HF**: High-frequency power; **LF/HF**: Low-frequency to high-frequency ratio; **TP**: Total power; **SDNN**: Standard deviation of normal-to-normal RR intervals; **RMSSD**: Root mean square of successive differences; **TINN**: Triangular interpolation of NN intervals; **NN50**: Number of successive RR interval pairs differing >50 ms; **pNN50**: Proportion of NN50; **HR**: Heart rate; **SBP**: Systolic blood pressure;

DBP: Diastolic blood pressure; **VAS**: Visual analogue scale; **NDI**: Neck Disability Index; **PPT**: Pressure pain threshold; **PDI**: Pain Disability Index; **Rand36-PF**: RAND-36 Physical Functioning subscale; **WAS**: Work Ability Score; **CHQ-12**: Chinese Health Questionnaire-12; **CLBP**: Chronic low back pain; **WAD**: Whiplash-associated disorder; **FDR**: False discovery rate; **NR**: Not reported; **Δ**: Change score.

Supplementary Table S8. Multivariable regression analyses examining associations across autonomic, psychosocial, and pain processing domains in individuals with persistent spinal pain.

Study (Year)	Outcome Variable	Predictor Variable	Domain	B [95% CI]	r_partial	p-value	Model R²	Significance
**Bandeira et al., 2021 [39]**	Δ Pain intensity	Kinesiophobia	Psychosocial	β = 0.37 [NR]	0.38	0.009	12.5%	Yes
		LF/HF (pretask)	Autonomic	β = -0.21[NR]	NR	0.13	12.5%	No
**Ansuategui Echeita 2022 [37]**	Lifting capacity	MPT (QST)	Pain processing	−0.00 [−0.00, −0.00]	-0.39	<0.01	58.3%	Yes
	Physical functioning	RMSSD	Autonomic	0.19 [0.01,0.37]	0.26	<0.05	41.7%	Yes
	Disability	VAS pain	Pain processing	1.36 [0.18,2.54]	0.29	<0.05	34.8%	Yes
	Δ Lifting capacity	ΔRMSSD	Autonomic	0.60 [0.24,0.96]	0.48	<0.01	38.0%	Yes
	Δ Disability	ΔCSI-A	Psychosocial	0.77 [0.01,1.53]	0.36	<0.05	13.3%	Yes
	Δ Physical functioning	ΔPCS	Psychosocial	-0.42 [-0.76, -0.07]	-0.38	<0.05	17.3%	Yes

Multivariable regression analyses examining associations across autonomic, psychosocial, and pain processing domains in individuals with persistent spinal pain. Only variables aligned with the predefined domains of interest are presented. Each predictor is reported separately, even when derived from the same multivariable model. Model R^2 represents the total explained variance of the final regression model. Statistical significance was determined based on reported p-values. Abbreviations: **B**: Unstandardized regression coefficient; **β**: Standardized regression coefficient; **CI**: Confidence interval; **r_partial**: Partial correlation coefficient; **RMSSD**: Root mean square of successive differences; **LF/HF**: Low-frequency to high-frequency ratio; **CSI-A**: Central Sensitization Inventory (Part A); **PCS**: Pain Catastrophizing Scale; **MPT**: Mechanical pain threshold; **VAS**: Visual analogue scale; **QST**: Quantitative sensory testing; **NR**: Not reported; **Δ**: Change score.
